# Gene Body Methylation Confers Transcription Robustness in Mangroves During Long-Term Stress Adaptation

**DOI:** 10.3389/fpls.2021.733846

**Published:** 2021-09-22

**Authors:** Yushuai Wang, Aimei Dai, Yiping Chen, Tian Tang

**Affiliations:** State Key Laboratory of Biocontrol and Guangdong Key Laboratory of Plant Resources, School of Life Sciences, Sun Yat-sen University, Guangzhou, China

**Keywords:** gene body methylation, expression variation, convergent evolution, stress adaptation, mangroves

## Abstract

Whether induced epigenetic changes contribute to long-term adaptation remains controversial. Recent studies indicate that environmentally cued changes in gene body methylation (gbM) can facilitate acclimatization. However, such changes are often associated with genetic variation and their contribution to long-term stress adaptation remains unclear. Using whole-genome bisulfite sequencing, we examined evolutionary gains and losses of gbM in mangroves that adapted to extreme intertidal environments. We treated mangrove seedlings with salt stress, and investigated expression changes in relation with stress-induced or evolutionarily-acquired gbM changes. Evolution and function of gbM was compared with that of genetic variation. Mangroves gained much more gbM than their terrestrial relatives, mainly through convergent evolution. Genes that convergently gained gbM during evolution are more likely to become methylated in response to salt stress in species where they are normally not marked. Stress-induced and evolutionarily convergent gains of gbM both correlate with reduction in expression variation, conferring genome-wide expression robustness under salt stress. Moreover, convergent gbM evolution is uncoupled with convergent sequence evolution. Our findings suggest that transgenerational inheritance of acquired gbM helps environmental canalization of gene expression, facilitating long-term stress adaptation of mangroves in the face of a severe reduction in genetic diversity.

## Introduction

Plants are constantly exposed to chronic or recurring environmental stresses across generations (Franks and Hoffmann, 2012; Lamke and Baurle, 2017). Environmentally-induced epigenetic changes such as DNA methylation and small RNAs may contribute to phenotypic variations by modifying gene expression. As a mechanism connecting the genome to its environment, epigenetics plays a dual role in variability by either increasing plasticity or suppressing variation. On one hand, the heritable epigenetic responses, also known as stress memory, may increase transgenerational phenotypic plasticity (Kinoshita and Seki, 2014; He and Li, 2018; Turgut-Kara et al., 2020). Increased variability might increase fitness and enable rapid adaptation to environmental fluctuations (Mirouze and Paszkowski, 2011; Wibowo et al., 2016; Chang et al., 2020). Epigenetics is thus often appreciated for its contribution to acclimation (Richards et al., 2017; Li et al., 2018; Liew et al., 2018). On the other hand, the most adaptive norm of reaction for many characteristics may be a constant phenotype buffered against environmental or genetic alteration, a phenomenon coined as canalization (Waddington, 1942a). In this scenario, epigenetics is thought to mediate phenotypic robustness *via* reducing gene expression variation (Wu et al., 2009). In comparison with promoting phenotypic plasticity, the canalizing role of epigenetics and its contribution to long-term adaptation is much less addressed.

In plants and animals, some genes have an increased level of methylated cytosines specifically in a CG sequence context, a phenomenon called gene body methylation (gbM) (Bewick and Schmitz, 2017). GbM is widespread and conserved across orthologs from divergent species despite its lability among tissues and individuals (Vining et al., 2012; Takuno and Gaut, 2013). Although whether or not gbM has a function has been controversial (Teixeira and Colot, 2009; Bewick and Schmitz, 2017), there is a hypothesis that gbM functions in stabilizing gene expression (Zilberman, 2017). Consistent with this hypothesis, experimental evidence has shown that gbM may regulate splicing (Yang et al., 2014; Wang et al., 2016) or prevent aberrant initiation of transcription (Bird, 1995; Horvath et al., 2019; Choi et al., 2020), albeit contradictory findings were also reported (Bewick et al., 2016). Meanwhile, a handful of comparative genomic studies have shown that gbM is associated with less variable gene expression both within and between species (Zilberman et al., 2008; Coleman-Derr and Zilberman, 2012; Steige et al., 2017; Takuno et al., 2017; Horvath et al., 2019; Seymour and Gaut, 2020). Recently, gbM was found to play a role in local adaptation and acclimatization in a reef-building coral (Dixon et al., 2018; Li et al., 2018; Liew et al., 2018). A reciprocal transplantation study of *Acropora millepora* reported that gbM disparity change between the high- and low-quality habitats, which reflects a shifting balance between expression of environmentally responsive and housekeeping genes, contributes to acclimatization (Dixon et al., 2018). However, its contribution to longer-term adaptation is still uncertain because between-population gbM differences did not align with plastic gbM changes but were mostly coincident with between-population genetic divergence, suggesting that genetic divergence is dominate in this case (Dixon et al., 2018). Gene body methylation also significantly reduces spurious transcription and transcriptional noise of highly expressed genes in *Stylophora pistillata*, providing corals with an additional mechanism to cope with environmental change (Liew et al., 2018).

Recent studies suggested that plants likely have different epigenetic responses in the face of short- and long-term environmental stresses. In annuals such as *Arabidopsis*, transient hyperosmotic stress memory is mainly due to changes in transposable element (TE) methylation (Wibowo et al., 2016), whereas ten-generation exposures to soil salinity promote accumulation of *de novo* methylated cytosines more frequently in genic regions (Jiang et al., 2014). Moreover, epigenetic memory in *Arabidopsis* can be lost in the absence of stress within a few generations (Jiang et al., 2014; Wibowo et al., 2016). In wild strawberry, acquiring a stable epigenetic memory requires repeated historical stress levels associated with heterogeneous environmental conditions while short-term acute stress did not result in significant epigenetic changes (De Kort et al., 2020). Therefore, extremophiles adapted to challenging environments for a long time may better serve the purpose of understanding the role of epigenetics in stress adaptation than model plants.

Thriving in the tropical and subtropical intertidal zones, mangrove trees represent an ideal model to elucidate how epigenetic mechanisms contribute to long-term stress adaptation. Mangroves exhibit transcriptional homeostasis under salt stress (Liang et al., 2012) and have fewer stress-responsive genes in their transcriptomes (Dassanayake et al., 2009) than glycophytes, suggesting that selection for robustness has reshaped gene expression profiles in these species. Furthermore, differential microRNA expression between mangroves and glycophytes is reminiscent of adaptive stress-responsive expression changes of these microRNAs in *Arabidopsis* (Wen et al., 2016), suggesting mangroves are capable of retaining inducible epigenetic changes in the long run. Last, adaptive convergence in sequence evolution among mangrove genomes has been well-studied. In mangrove genomes, about 400 genes have experienced convergence over the background level of convergence in the non-mangrove relatives (Xu et al., 2017); convergent reduction in TE numbers was also evident in these species (Lyu et al., 2018). It is thus possible to disentangle the roles of epigenetic changes and genetic variation in mangrove stress adaptation.

Here we report that three main mangrove taxa (i.e., *Avicennia marina*, *Rhizophora apiculata*, and *Sonneratia alba*) acquired *de novo* gbM genome-wide since their divergence from their respective common ancestors. GbM convergently acquired by mangroves may represent salt-induced epigenetic changes evolved under selection and correlates with homeostatic expression profiles under high salinity. Nevertheless, genes with convergently acquired gbM show little overlap with loci showing signatures of convergent sequence evolution. Our results indicate that epigenetics plays an independent role in expression robustness. This may allow accelerated accumulation of genetic variation, facilitating long-term stress adaptation.

## Materials and Methods

### Bisulfite Sequencing and Methylome Analyses

Whole genomes of mangroves *Avicennia marina*, *Rhizophora apiculata*, and *Sonneratia alba* have been sequenced and annotated previously (Xu et al., 2017; He et al., 2018; Lyu et al., 2018). The current-year leaves at branch tips were collected for the three mangrove species each with three replicates from Qinlan Harbor, Hainan, China. Genomic DNA was extracted using a modified CTAB protocol (Doyle and Doyle, 1987). Whole genome bisulfite sequencing (BS-seq) was conducted using 100 or 150 bp paired-end reads as previously described (Wang et al., 2018). BS-seq data from *Oryza sativa*, *Mimulus guttatus*, *Populus trichocarpa* and *Eucalyptus grandis* were downloaded from NCBI Short Read Archive database (Supplementary Table 1) and reanalyzed using the same workflow as for mangroves. Genome sequences and annotations of the non-mangrove species were retrieved from Phytozome v.12.1^1^.

After trimming of low-quality bases using Trimmomatic v.0.32 (Bolger et al., 2014), BS-seq reads were mapped to the appropriate genome using Bismark v.0.16.3 (Krueger and Andrews, 2011) with default parameters. Only uniquely mapping reads were retained. Bisulfite conversion rates were estimated based on reads that uniquely aligned to the chloroplast or the lambda genome if available (Supplementary Table 1). Cytosines were called as methylated (False discovery rate, FDR < 0.05) using a binomial test employing the conversion rate as the expected probability followed by Benjamini–Hochberg correction (Benjamini and Hochberg, 1995). Only cytosines with consistent methylation status in at least two replicates were retained. Cytosines covered by fewer than three sequencing reads were discarded in further analyses. Methylation level of a given genome was calculated as the proportion of methylated cytosines among total cytosines genome wide by sequence context (CG, CHG and CHH, where H is A, T, or C).

### Defining Body-Methylated Genes

A probabilistic approach was used to identify body-methylated genes as described previously (Takuno and Gaut, 2012). The methylated and total cytosines were counted for the coding sequence (CDS) of the primary transcript of each gene by sequence context (CG, CHG and CHH). A binomial test was applied to assess whether the abundance of methylated cytosines in a gene significantly departs from the abundance of methylated cytosines genome wide using the Benjamini–Hochberg FDR correction for each sequence context separately. Each species had its own basal value for the binomial test. Body-methylated (BM) genes were identified as those that have FDR < 0.05 for CG context and FDR > 0.05 for the CHG and CHH context. Similarly, we considered genes with significantly more methylated cytosines at CHG (FDR < 0.05 for CHG and FDR > 0.05 for CHH context) or CHH context (FDR < 0.05 for CHH context) as mCHG or mCHH genes, respectively (Niederhuth et al., 2016; Bewick and Schmitz, 2017). Genes without enrichment of methylated cytosines in any of the three sequence contexts (all FDR > 0.05) were considered unmethylated (UM). Only genes with sufficient cytosine content (≥20 CG, CHG or CHH sites) were considered in this framework (Niederhuth et al., 2016). All statistical analyses were conducted using R v.3.1.3^2^.

### Ortholog Identification and Sequence Analyses

Orthologous gene clusters of the seven species in our survey were constructed by OrthoMCL (Li et al., 2003) using the primary amino acid sequence of each gene with ≥40% identity, e-value cutoff of 1e-10 and a default inflation value of 1.5. Genes shorter than 50 amino acids were discarded. Only clusters with at least one gene per species were used in the following analyses. If more than one gene of any species was in an OrthoMCL orthologous gene cluster, the one that showed the highest similarity to all other genes of the cluster as assessed with BLASTn (NCBI) was retained (Xu et al., 2017).

### Estimation of gbM Gain and Loss Rates

Based on the phylogenetic tree of the seven species in our set, we first inferred gbM status (BM or UM) at each internal node for the 1:1 ortholog gene clusters. Ortholog gene clusters that contained CHG or CHH genes were excluded from all further analyses in this study. Using a maximum parsimony method, the inference started from each species pair of mangroves and non-mangroves, and then extended hierarchically along branches of the phylogenetic tree until gbM status of the most recent ancestor of all three species pairs was resolved. The gbM status of the rice gene was considered as the ancestral state whenever the ancestral gbM status of all three pairs could not be resolved by applying the maximum parsimony method to only mangroves and in-group non-mangrove species. Given the estimated ancestral gbM status at each internal node, we counted the number of genes that gained or lost gbM in each branch of the phylogenetic tree. The gain or loss rate of gbM for a given branch was calculated as the number of lineage-specific gains or losses of BM genes divided by the number of UM or BM genes at the most closely related internal node using a maximum likelihood method (Takuno et al., 2017).

### Inference of gbM Convergence and GC Site Convergence

To infer gbM convergence in mangroves, we applied a modified Convergence at Conservative Sites (CCS) method (Xu et al., 2017) to gbM status of the 1:1 ortholog gene clusters. Given the estimated ancestral gbM status as describe above, we inferred gbM convergence for either mangroves or non-mangroves if at least two of the three mangroves or non-mangrove species shared the derived BM status that is different from the ancestral state of UM (Supplementary Figure 1). GbM status across species was visualized using UpSet (Lex et al., 2014). The modified CCS method was also applied to methylation status (methylated or unmethylated) of conservative CG sites at the 1:1 ortholog clusters to infer convergence of DNA methylation at individual CG sites. We required the CG sites analyzed to be conserved across all seven species.

### Plant Growth and Salt Treatment

Propagules of *A. marina* and *R. apiculata* were collected from Qinlan Harbor, Hainan, China where the average seawater salinity in mangrove swamps is about 15 ppt (Zheng et al., 2018), and planted in culture pots containing a mixture of sand and nutritive soil under a natural photoperiod. The seedlings were freshwater-irrigated until they produced more than four true leaves, usually growing to about 30 cm tall. For salt treatment, three seedlings were cultured in pots that were half-submerged in 500 mM NaCl and watered everyday with the same solution for 7 days. Three untreated seedlings were cultured in freshwater in parallel as controls.

### Identification of Salt-Induced Differentially Methylated Positions and Differentially Methylated Genes

Leaves were collected from three biological replicates each of the salt-treated and untreated seedlings of *A. marina* and *R. apiculata*. DNA isolation and BS-seq analysis were conducted as described above. To identify differentially methylated position (DMPs), we applied a generalized linear model on individual cytosine sites (≥5 reads). The general formula used was glm (methylated, non-methylated ∼ treatment + individual, family = “binomial”), where “methylated, non-methylated” was a two-column response variable denoting the number of methylated and non-methylated reads at a particular position. For predictor variables, “treatment” denoted salt-treated or untreated conditions, while “individual” denoted different replicates. *P* values of the “treatment” factor were used for estimation of genome-wide FDRs using the Benjamini–Hochberg procedure (Benjamini and Hochberg, 1995). Cytosine sites with FDR < 0.05 were considered as DMPs. To identify differentially methylated gene (DMGs), we first identified gbM for salt-treated and untreated samples separately using the probabilistic approach described above, and then considered genes displaying changes between gbM and UM states after salt treatment as salt-induced DMGs.

### RNA-Seq Analyses

Total RNA was extracted from leaves of three each of the salt-treated and untreated replicates of *A. marina* and *R. apiculata*, and used for RNA-seq as previously described (Wang et al., 2018). After quality control using Trimmomatic v.0.32 (Bolger et al., 2014), clean reads were mapped to the appropriate repeat-masked genomes using Bowtie (Langmead et al., 2009) allowing ≤2 mismatches. The expression level of each gene was analyzed using HTSeq v.0.6.1 (Anders et al., 2015) with the parameter: -s no and normalized as Reads Per Kilobase per Million mapped reads (RPKM). Genes with no reads mapping in all sequencing libraries were discarded in further expression analyses. Differentially expressed genes were identified with DESeq2 (Love et al., 2014) requiring Benjamini–Hochberg multiple testing corrected (Benjamini and Hochberg, 1995) FDR < 0.05 and ≥2 fold change.

### Measurement of Expression Difference

We measured expression difference between salt-treated and control samples using the method described by Takuno et al. (2017). Briefly, gene expression level of three replicates within treated or control samples were averaged and then normalized to a mean of 0 and variance of 1 within each group. The absolute expression difference between the treated and control samples for each species was calculated as | *e1*-*e2*|, where *e1* and *e2* are the normalized expression levels in treated and control samples, respectively. Gene expression level log10 RPKM ≤ 0.01 were ignored in this analysis. All statistical analyses were conducted using R v.3.1.3.

### Gene Ontology and Kyoto Encyclopedia of Genes and Genomes Analyses

We obtained Gene Ontology (GO) terms and kyoto encyclopedia of genes and genomes (KEGG) annotations of mangrove unigenes from literature (Xu et al., 2017). The GO and KEGG assignment of *R. apiculata* was used to represent the ortholog clusters. GO term enrichment analyses of convBMM genes were carried out using WEGO2 (Ye et al., 2006) with default settings. On the basis of the KEGG annotation, Fisher’s exact test and Benjamini–Hochberg correction (Benjamini and Hochberg, 1995) were carried out to test for statistical significance of specific pathway enrichment.

## Results

### Genome-Wide Methylation Distribution in Mangrove and Non-mangrove Lineages

**FIGURE 1:**
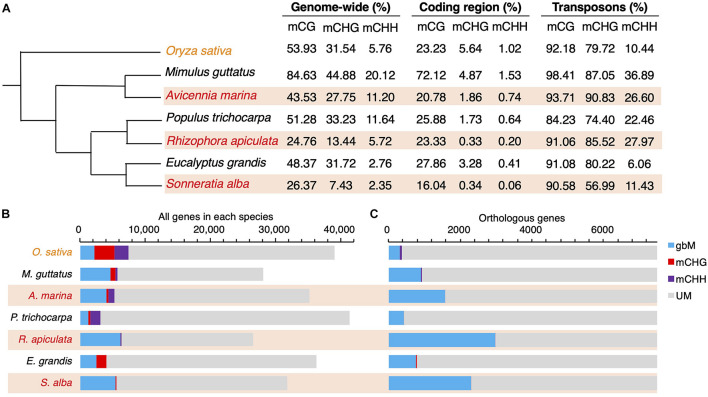
Genome-wide methylation distribution across mangrove and non-mangrove lineages. Tree topology is adopted from Xu et al. (2017). **(A)** Methylation levels measured as a percentage of methylated cytosine residues genome wide or in coding regions and transposons are shown by sequence context. **(B)** Bar plot shows the relative abundance of BM, mCHG, mCHH (where H is A, T, or C), and UM genes genome wide or in **(C)** 1:1 ortholog clusters for each species. BM, genes body-methylated in the CG context; mCHG, in the CHG context; mCHH, in the CHH context; UM, unmethylated genes. Mangrove species are indicated with shadows in orange. Species are organized according to their phylogenetic relationships.

Plant cytosine DNA methylation occurs in three sequence contexts: CG, CHG, and CHH (where H = A, C, T) (Henderson and Jacobsen, 2007). We compared genome-wide methylation distribution in leaves between three species pairs of mangroves and non-mangroves (*A. marina vs. Mimulus guttatus*, Ama-Mgu; *R. apiculate* vs. *Populus trichocarpa*, Rap-Ptr; and *S. alba vs. Eucalyptus grandis*, Sal-Egr), using *Oryza sativa* as an outgroup (Figure 1). The divergence times for Ama-Mgu, Rap-Ptr and Sal-Egr were about 64, 92 and 66 Mya, respectively, as estimated by TimeTree database (Hedges et al., 2015). This design of species comparisons is the same as that used previously in a study on mangrove sequence convergence (Xu et al., 2017) except that we used *M. guttatus* for the availability of methylome data (Supplementary Table 1). We preformed whole-genome bisulfite sequencing (BS-seq) with an average coverage of 25 × per individual on leaves of the three mangroves each with three replicates and retrieved published BS-seq data from leaves of the non-mangroves (Supplementary Table 1; Stroud et al., 2013; Niederhuth et al., 2016). Pairwise correlation analysis of the methylation levels among replicates showed good reproducibility in all three sequence contexts for all the mangrove species (Pearson’s correlation, *r* = 0.94–0.99, all *P* < 2.2 × 10^–16^; Supplementary Figure 2). Given their history of stress adaptation, we expected that mangroves have acquired more methylated CG sites than their non-mangrove relatives since environmental stress is known to accelerate the accumulation of epimutations (Jiang et al., 2014).

However, when comparing the two groups of species, we found that mangroves exhibit slightly lower genome-wide methylation levels on average than their non-mangrove relatives in all three contexts (the difference was only significant for the CHG context: two-tailed *t*-test, *P* < 0.05; Figure 1A). Methylation levels averaged separately for mangroves and non-mangroves in coding regions and TEs were largely comparable between the two species groups in all three contexts (Figure 1A and Supplementary Table 2), suggesting the observed differences in genome-wide mCHG levels are mainly attributable to the reduction of TE numbers in mangroves (Lyu et al., 2018).

### Accelerated Accumulation of gbM in Mangrove Lineages

We classified genes into one of the four categories: CG body methylated (hereafter BM), mCHG, mCHH, and unmethylated (UM), as previously described (Takuno and Gaut, 2012). The vast majority of genes are in the UM group and ∼2% belong to mCHG or mCHH (Figure 1B). Mangroves indeed possess many more BM genes (5,203 or 17.2%, averaged across species hereafter) than the non-mangroves we examined (2,670 or 8.1%; Figure 1B). To enable a direct comparison between species, we identified a set of 7,488 1:1 ortholog clusters among all seven species using OrthoMCL (Li et al., 2003) and a previously-described method (Xu et al., 2017). This subset yielded a distribution of methylated sites similar to the one we see in the whole genome (Figure 1C) and the difference is significant (two-tailed *t*-test, *P* < 0.05).

**FIGURE 2:**
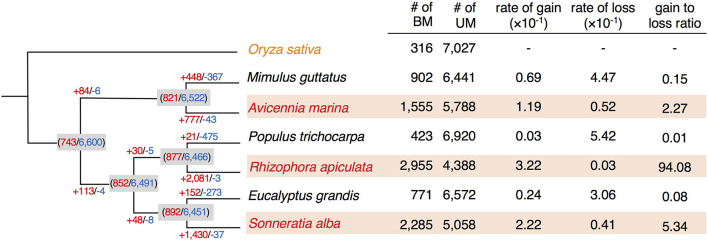
gbM gain and loss across orthologs of mangroves and non-mangrove species. The estimated numbers of gain (red) and loss (blue) of gbM at each internal node are given in parentheses. The estimated numbers of gbM gain and loss in each branch are indicated with plus in red or with minus in blue, respectively. BM, CG body-methylated genes, UM, unmethylated genes.

We further estimated the rates of gbM gain and loss along each branch of the phylogeny based on 7,343 1:1 ortholog clusters (after excluding those containing mCHH or mCHG genes). These 1:1 ortholog clusters included all the orthologs (7,274), inparalogs (5,494), and co-orthologs (4,766) identified by OrthoMCL (Li et al., 2003). In cases where multiple paralogs were present within a species, only the one most similar to other genes in the cluster was retained because the method used to detect convergent evolution only considers cases of one-to-one orthology. However, taking into account the change of methylation status among paralogs, only about 0.6–1.8% of these gene clusters had methylation status differing from that assigned to the 1:1 orthologs (Supplementary Table 3). Consequently, the overall methylation patterns reported here would not be substantively altered had we included all paralogs. As shown in Figure 2, mangroves exhibit remarkably more gbM gains (1,429) but fewer losses (28) than their non-mangrove relatives (gain: 207 and loss: 371; two-tailed *t*-test, *P* < 0.05). Similarly, the estimated rate of gbM gain is one or two orders of magnitude higher in mangroves than in their non-mangrove counterparts (two-tailed *t*-test, *P* < 0.05, Figure 2), while the rates of loss show an opposite pattern (Figure 2). The gain to loss ratio ranges from 2.27 to 94.08 in mangroves, in striking contrast to an average of 0.08 in non-mangroves (Figure 2). It is possible that the strong selection history of domesticated rice may introduce bias to the estimation of gbM gain and loss rates. We therefore used *Setaria viridis*, a species of grass with both genome and methylome available (Niederhuth et al., 2016) as the outgroup, and conducted the same analyses. Once again, we found significantly higher gbM gain rate (two-tailed *t*-test, *P* < 0.05, Supplementary Figure 3) and lower gbM loss rate (two-tailed *t*-test, *P* < 0.01, Supplementary Figure 3) in mangroves in comparison with their non-mangrove counterparts. Taken together, these results strongly suggest that accumulation of methylated genes has been accelerated repeatedly across mangrove lineages since the split of mangrove and non-mangrove species from their common ancestor. Hereafter, we present the results using rice as the outgroup, which allows us to compare epigenetic and genetic evolution directly since rice was also used to study convergent evolution of nucleotide sequences among mangroves (Xu et al., 2017).

### Convergent Evolution of Newly Acquired gbM in Mangroves

We speculate that the evolutionarily acquired gbM across mangrove lineages may represent induced epigenetic changes maintained by natural selection. If so, we expect that a substantial fraction of them have undergone convergent evolution. To identify such loci in the three mangroves, we applied the convergence at conservative sites (CCS) method (Xu et al., 2017) to gbM statuses of our list of 7,343 orthologs. Given the symmetric design of mangrove and non-mangrove pairs, convergent gain of gbM status can be inferred for either mangroves or non-mangrove species if at least two of the three other species share a derived body methylation character that is different from the ancestral state (see section “Materials and Methods” and Supplementary Figure 1). It should be noted that the identification of convergent gbM gain does not require a gene to be gbM in all three species of a given group. As the spontaneous epimutation rates are known to be extremely high (∼10^–4^ per site per generation) and biased to methylation loss (van der Graaf et al., 2015), the loss of gbM in a certain lineage would not be seldom.

**FIGURE 3:**
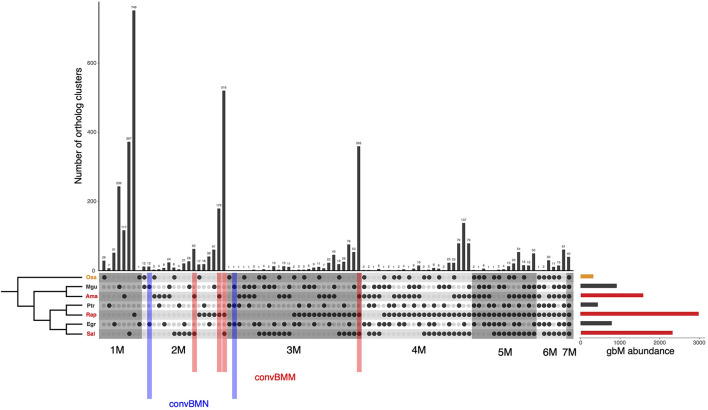
Conservation of gbM status across orthologs of the three mangroves and their non-mangrove relatives. The UpSetR plot shows all intersecting sets of the gbM status between species examined. The number of genes in each intersecting set is given at the top of the histogram. In each species, the gbM status is indicated with filled or open circle for BM and UM genes, respectively. Orthologous genes were grouped by their conservation of gbM and labeled from 1 to 7 plus “M,” indicating the number of species where the gbM status is conserved. Bar plot displays the abundance of BM genes in each species. convBMM, BM genes with convergence of gbM specific to mangroves; convBMN, BM genes with convergence of gbM specific to non-mangroves. Osa, *Oryza sativa*; Mgu, *Mimulus guttatus*; Ptr, *Populus trichocarpa*; Egr, *Eucalyptus grandis*; Ama, *Avicennia marina*; Rap, *Rhizophora apiculata*; Sal, *Sonneratia alba*.

Among the 7,343 orthologs, 743 were inferred to be BM genes and 6,600 to be UM genes in the common ancestor of the three species pairs (Figure 2). 757 (10.3%) and 355 (4.8%) genes convergently gained gbM in two or three mangroves, and we refer to these genes as “convBMM” (Figure 3). In contrast, only 12 (0.2%) loci gained gbM in two and only one gained it in all three non-mangrove controls. We refer to these genes as “convBMN” (Figure 3). The observed convergent evolution of gbM in mangroves is significantly higher than the background level in the non-mangrove controls (Fisher’s exact test, both *P* < 2.2 × 10^–16^). The asymmetric pattern of gbM convergence indicates that a large fraction of evolutionarily acquired gbM in mangroves is favored by selection and thus likely beneficial.

Interestingly, convergent evolution of methylation at the whole-gene level is uncoupled from evolution of individual methylated CG sites. Only eight out of the 8,987 conserved CG sites among all species in our sample show convergent gains of CG methylation in mangroves, whereas the CCS method detected convergence at 138 sites that gained CG methylation in non-mangroves (χ^2^ test, *P* < 0.001).

### Genes With Mangrove-Specific Convergent gbM Preferentially Gained It in Response to Salt Stress

**FIGURE 4:**
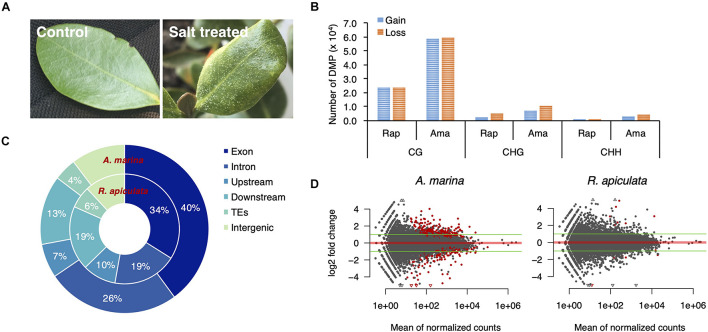
Salt-induced changes of DNA methylation and gene expression in *A. marina* and *R. apiculata*. **(A)** Salt-treated and untreated leaves of *A. marina*. **(B)** The number of differentially methylated positions (DMPs) in response to salt treatment are shown by sequence context (i.e., CG, CHG and CHH, where H is A, T, or C). **(C)** The genomic distribution of CG-DMPs. **(D)** Fold changes of gene expression between salt-treated and untreated samples plotted against the averaged expression levels (the average of reads counts normalized by size factor) between them. The red points indicate genes with significant (Benjamini–Hochberg FDR < 0.05) differential expression.

Coping with salt marshes, mangroves have developed a variety of strategies to adapt to extreme saline environments. While *A. marina* secretes excess salt *via* salt glands on leaf surfaces, *R. apiculata* and *S. alba* exclude salts from its roots (Scholander et al., 1962). As the latter two species share a similar salt tolerance mechanism and are more closely related with each other than with *A. marina*, we chose the more diverged *A. marina* and *R. apiculata* to explore the potential role of gbM gains in mangrove adaptation. We planted propagules of *A. marina* and *R. apiculata* derived from wild plants that naturally grow in coastal saline water with fresh water. Seedlings were treated with 500 mM NaCl, about two times of the salinity in natural habitats, for 7 days and the untreated plants were used as a control. *A. marina* wilted and secreted salts on leaves after treatment (Figure 4A), indicating that the salt levels used were sufficiently high to stress the plants.

We analyzed changes in DNA methylation and gene expression between leaves of treated and untreated plants using BS-Seq and RNA-seq, each with three biological replicates per treatment. Using a generalized linear model, we detected 14.4 × 10^4^ and 5.6 × 10^4^ differentially methylated positions (DMPs; FDR < 0.05), i.e., individual cytosines with a significantly altered methylation frequency in response to salt, out of the 152.7 million cytosines in *A. marina* and 81.1 million in *R. apiculata* covered by more than five reads (Supplementary Table 4). The vast majority of DMPs occurred in the CG context (Figure 4B). These salinity-associated CG-DMPs arose more frequently in genic than non-genic regions (hypergeometric test, both *P* < 0.001, Figure 4C), similar to previous findings in *Arabidopsis* (Jiang et al., 2014). In comparison to the control, the salt-induced gains and losses of methylation were comparable in both species, while slightly more methylation losses were observed particularly in the CHG context (Figure 4B).

If the convergent acquisition of gbM in mangroves was originally induced when woody plants independently invaded intertidal zones, we expect genes that have lost convergent gbM in some mangrove species (i.e., genes with conserved gbM in two mangrove species only) are inclined to regain gbM in response to salt stress therein. Using the binomial test described above, we identified body-methylated genes in salt-treated and control plants separately (FDR < 0.05), and found that 672 genes in *A. marina* and 367 in *R. apiculata* changed their methylation status in response to salt (Table 1). Among genes classified as unmethylated in the *A. marina* control plants, induced gbM gains in the salt-treated plants are enriched for genes that were identified as convergently body-methylated in mangroves (convBMM in Figure 3) compared to all other UM loci (11.7 vs. 1.1%, χ^2^ test, *P* < 10^–5^, Table 1). The same pattern emerges in *R. apiculata* (19.8 vs. 0.9%, χ^2^ test, *P* < 10^–5^, Table 1). These results suggest that convergent acquisition of gbM in mangroves is induced by environmental stress. convBMM genes in *R. apiculata* also tend to maintain the gbM state under stress (χ^2^ test, *P* < 0.01, Table 1), coincident with the strikingly high ratio of gbM gain rate to loss rate observed in this species (94.08 in Figure 2).

**TABLE 1:** Salt-induced methylation changes in genes convergently gaining methylation in mangroves.

Species	Methylation type	Gene clusters	gbM status		% of DMGs	χ^2^ test (*P* value)
			Unchanged	Changed		
*A. marina*	BM	convBMM	541	36	6.7	>0.05
		others	3,337	269	8.1	
	UM	convBMM	479	56	11.7	<0.001
		others	28,875	311	1.1	
*R. apiculata*	BM	convBMM	984	13	1.3	<0.01
		others	4,817	155	3.2	
	UM	convBMM	96	19	19.8	<0.001
		others	20,243	180	0.9	

*BM, body-methylated; UM, unmethylated; convBMM, convergently body-methylated in mangroves; others, other BM or UM genes in separate species.*

We wondered whether covBMM genes preferentially gaining salt-induced gbM is reminiscent of previous stress response or labile plasticity. To answer this question, we compared the global gbM patterns in control seedlings that underwent one generation without salt stress and plants in nature that are exposed to saline environments for many generations. More than 92.9% of the gbM genes identified in plants of *A. marina* (3,772 out of 4,060) or *R. apiculata* (5,736 out of 6,177) in nature were also found to be gbM genes in control seedlings, indicating that the gbM status is stable for at least one generation in the absence of salt stress. When we grouped genes by whether their gbM states are identical between control seedlings and the plants in nature, only those with consistent gbM status showed the preference in gaining salt-induced gbM for convBMM genes in both species (χ^2^ test, *P* < 0.05; Supplementary Table 5). These results suggested that covBMM genes have the stress memory of gaining gbM in response to salt stress and the gbM status of these genes are likely to be heritable epigenetic changes evolved under selection.

Whole transcriptome expression profiles between salt-treated and untreated samples identified only 319 (209 up-regulated and 110 down-regulated) differentially expressed genes [FDR < 0.05 and log2(fold change) ≥ 1] in *A. marina* and 16 (10 up-regulated and six down-regulated) in *R. apiculata* (Figure 4D). Like a previous report in the mangrove *Ceriopsis tagle* (Liang et al., 2012), this result suggests that the current mangrove transcriptomes evolved robustness to environmental salinity. Four differentially-expressed genes (two up-regulated and two down-regulated) lost their gbM status in *A. marina* and no change of methylation status was found for differentially-expressed genes in *R. apiculata* under salt treatment. Furthermore, we observe no correlation between changes in DNA methylation and changes in gene expression in either species (Pearson’s correlation, *P* > 0.05; Supplementary Figure 4). This suggests that methylation changes play little role, if any, in altering expression levels in response to salt.

### Gains of gbM Correlate With Reduction of Stress-Responsive Expression Variation

**FIGURE 5:**
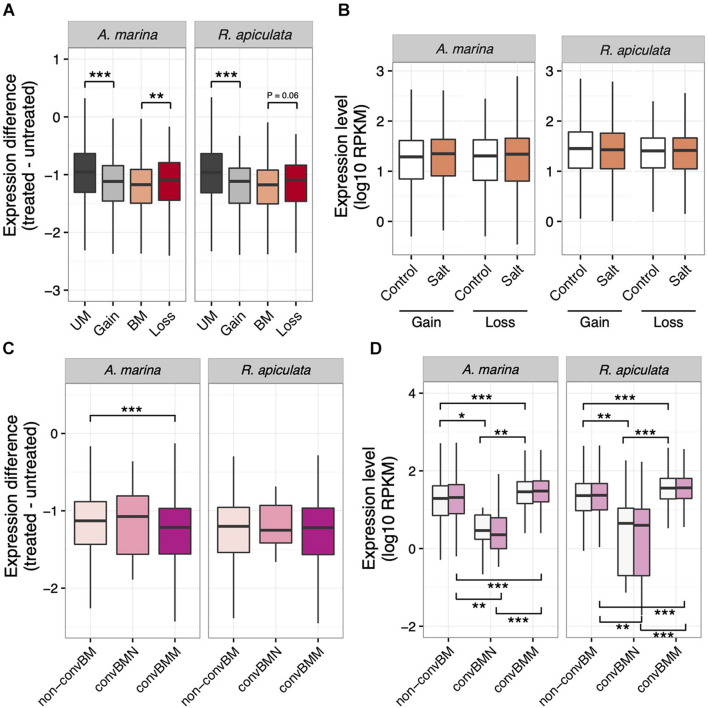
Expression patterns in relation to gbM status in *A. marina* and *R. apiculata* under salt stress. **(A)** Absolute expression difference of genes that gained or lost gbM in response to salt stress. **(B)** Expression level of genes that gained or lost gbM in response to salt stress. **(C)** Salt-responsive absolute expression differences in relation to gbM convergence. **(D)** Expression levels in relation to gbM convergence in salt-treated (pink) and control samples (white). BM, CG body-methylated genes; UM, unmethylated genes. convBMM, BM genes with convergence of gbM specific to mangroves; convBMN, BM genes with convergence of gbM specific to non-mangroves; non-convBM, BM genes with gbM conserved in two or three species but not convergently evolved in either mangroves or non-mangroves. Significance was determined by the Mann–Whitney *U* test: *P < 0.05, **P < 0.01, ***P < 0.001. The middle bars in the boxes represent the median; the top and bottom of the boxes indicate interquartile ranges, and whiskers denote the range.

More changes in methylation than in expression under salt stress suggests that epigenetics may help buffer expression variation against environmental challenges. We therefore tested whether salt-induced gains of gbM resulted in a reduction of salt-responsive variance in transcription. We normalized average read counts (log10 RPKM) across replicates of each treatment and calculated the absolute difference (Takuno et al., 2017) between normalized expression levels in treated and untreated samples for each species. Indeed, genes that became methylated under high salinity exhibited lower expression difference than UM genes that did not change their gbM across conditions (Mann–Whitney *U* test, *P* < 0.001; Figure 5A). Interestingly, genes that lose gbM in response to salt treatment are less buffered compared to genes that maintain methylation irrespective of salt levels (Mann–Whitney *U* test, *P* < 0.05; Figure 5A). In contrast, no significant changes in expression level were found for genes that gained or lost gbM under salt stress (Figure 5B). This pattern is evident in both *A. marina* and *R. apiculata* (Figure 5B), indicating that stress-induced gains of gbM correlate with reduced gene expression variation.

Since gbM gain under stress treatment seems to stabilize gene expression, we wondered whether selection favors convergent gbM gains in mangroves for increased transcription robustness against environmental stress. In both *A. marina* and *R. apiculata*, the average expression change under salt treatment is smaller for orthologs that are methylated in more than one species than for loci that are either unmethylated or have gained gbM in one lineage only (Mann–Whitney *U* test, all *P* < 0.001, Supplementary Figure 5). This result suggests that evolutionary gain of gbM has a similar homeostatic effect and its efficacy correlates with gbM conservation. We therefore separately analyzed genes that convergently gained gbM in mangroves (convBMM in Figure 3) in comparison with genes that exhibited the same level of gbM conservation (i.e., body-methylated in two or three taxa) but gained gbM convergently in non-mangroves (convBMN) or in neither group (non-convBM).

As expected, convBMM genes were relatively resistant to the influence of salt stress in *A. marina* (Mann–Whitney *U* test, *P* < 0.001; Figure 5C) while their expression was also higher overall (Mann–Whitney *U* test, all *P* < 0.01, Figure 5D). We were unable to replicate the pattern in *R. apiculata*, however, likely due to low general expression differences. Thus, it appears that convergent gains of gbM in mangroves were indeed selected for robust expression against environmental stress.

### Convergent Methylation and Sequence Evolution Are Uncorrelated

A previous study identified 232 genes that show signs of convergent evolution in mangroves at the DNA sequence level (Xu et al., 2017). We therefore asked whether the same set of genes underwent convergent epigenetic evolution. We find that only 38 genes are in common between the two groups, suggesting that different biological processes are targeted by selection for genetic and methylation changes (Supplementary Figure 6A and Supplementary Table 6). This is further corroborated by the observation that genes with signatures of sequence convergence are not more likely than average to gain methylation in response to salt stress (χ^2^ test, *P* > 0.05 in both species; Supplementary Table 7).

Gene ontology and KEGG analyses detected no enrichment in functional categories among loci convergently gaining gbM. The majority of these genes play essential functions such as “catalytic activity” (GO:0003824) and “binding” (GO:0005488) while only a small fraction of them are involved in “response to stimulus” (GO:0051716) (Supplementary Figure 6B). Similarly, only three out of the 38 genes that convergently gained gbM and also convergently evolved at the DNA sequence level are involved in “response to stimulus” (Supplementary Table 6). These are *Ethylene response 2* (*ETR2*) that negatively regulates ethylene response in *Arabidopsis* (Hua and Meyerowitz, 1998), and the mitochondrial *ATP-dependent proteases FtsH protease 3* (*FtsH3*) and *Long protease homolog 1* (*Lon1*) that function in homeostasis of mitochondrial proteins (Janska et al., 2010; Li et al., 2017; Supplementary Table 6).

## Discussion

Ever since Waddington proposed the concept of epigenetics (Waddington, 1942b), its role in evolution has been hotly debated. Attempting to understand the contribution of epigenetics to long-term adaptation, we jointly analyzed genome-wide dynamics of gbM in response to environmental stimulus in mangroves and during their adaptation to coastal environments. We find accelerated gains of gbM in the mangrove lineages. A large fraction of these gbM acquisitions underwent convergent evolution, strongly implying they are adaptive. Moreover, convergently gained gbM during evolution are prone to salt-induced methylation gains in species where they are normally not marked, suggesting that the acquisition events are likely triggered by environmental cues. Our findings thus highlight that induced methylation changes can be substrates for natural selection and fuel long-term stress adaptation.

Interestingly, combinatorial analyses of methylomes and transcriptomes under salt treatment (Figures 4, 5) suggest that gbM mediates environmental canalization of gene expression in mangroves. The long-standing, predictably stressful environments may put strong selective pressure on mangroves in favor of expression robustness. Consistent with this speculation, mangroves also convergently lowered the representation of stress-responsive genes in their transcriptomes (Dassanayake et al., 2009) and eliminated transposable elements (Lyu et al., 2018). In contrast to its canalizing role in long-term adaptation, gbM involved in short-term adaptation appears to mediate a reaction norm allowing different optimal gene expression in line with environmental cues. This is evident from the study of coral transplantation, in which adaptive plastic changes in gbM correlate with upregulation of environmentally responsive genes in lower-quality habitats, whereas they correlate with upregulation of housekeeping genes in higher quality locations (Dixon et al., 2018).

The adjustment of balance between expression of housekeeping and environmentally responsive genes, proposed as an ecological role of gbM (Dixon et al., 2018), is pertinent to the interplay between expression robustness and variability. Environmentally responsive genes are characterized by high expression noise while housekeeping genes, often highly expressed, are transcriptionally stable (Lopez-Maury et al., 2008). It is thus significant that genes that convergently gained gbM in mangroves are mainly housekeeping. These results are consistent with previous findings that body-methylated genes usually perform housekeeping functions (Takuno and Gaut, 2012, 2013). These genes also exhibit a higher level of expression than control genes with the same level of gbM conservation (Figure 5D). It is unclear whether these directional changes in gene expression are selected for, or are just byproducts of selection for increased stability. We prefer the latter explanation given that induced changes in methylation show no correlation with changes in expression level (Figure 5B and Supplementary Figure 4). Our results together with a previous study (Dixon et al., 2018) indicate that gbM plays a systemic role in keeping reaction norms in tune with needs dictated by selection, promoting adaptation to both long- and short-term environmental changes.

In contrast to previous studies in *Arabidopsis* (Dubin et al., 2015; Meng et al., 2016) and corals (Dixon et al., 2018), where adaptive epigenetic changes are often tightly associated with genetic variation, there is little overlap between genes convergently acquiring methylation and loci exhibiting signatures of convergent DNA sequence evolution in mangroves. This result indicates that selection acting on epigenetic variation favors a cohort of genes differing from those favored by selection acting on genetic variation. Then, how do epigenetic changes interact with genetic variation to reconcile robustness and evolvability during long-term stress adaptation?

An intriguing possibility is that expression robustness encoded by epigenetics will allow for the silent accumulation of cryptic genetic variation while canalizing the optimal phenotype. Theoretical work has shown that epigenetic variants, as long as some are beneficial and heritable, can allow the population to adapt quickly by finding a local maximum in the absence of genetic diversity and subsequently evolve under stabilizing selection until an even fitter phenotype that is encoded by genetic mutations emerges (Klironomos et al., 2013). Such effects of epigenetics, however, do not require that the epigenetic marks be part of a genetically encoded adaptive plastic response (Klironomos et al., 2013). Given the extreme paucity of genetic variation in mangroves (Guo et al., 2018), the potential interplay between the acquired gbM states and the standing genetic variation might have sped up long-term adaptation of mangroves compared to cases where natural selection acts only on genetic variation.

It is interesting that mangroves experienced accelerated accumulation of gbM despite a lack of concomitant increase in spontaneous epimutation rates. This is evident by the abundant gbM but relatively low genome-wide methylation levels in mangroves compared to non-mangroves. A possible explanation is that the reduction in TE numbers (Lyu et al., 2018) decreases genome-wide methylation levels (Figure 1A) and consequently decreases the threshold for gbM detection. This hypothesis is compatible with the observed positive correlation between DNA methylation levels and genome size (Alonso et al., 2015; Niederhuth et al., 2016; Vidalis et al., 2016) which is mainly attributable to TE content variation in plants (Niederhuth et al., 2016; Takuno et al., 2016; Vidalis et al., 2016). Consistent with this hypothesis, we found *A. marina* is a bit of an outlier (with lower gbM than *M. guttatus* overall), as TE reduction in *A. marina* compared with *M. guttatus* (31.4 vs. 42.2%) was less severe than that between *R. apiculata* and *P. trichocarpa* (17.1 vs. 40.5%) or between *S. alba* and *E. grandis* (11.0 vs. 40.3%) (Lyu et al., 2018). However, further evidence is in need to test whether the association between gbM abundance and TE contents is a general phenomenon.

## Conclusion

Plants in nature are continuously exposed to diverse environmental challenges. Stress adaptation occurs on multiple time scales, including short-term response during a lifetime and longer-term responses across generations. Our work demonstrates that acquired gbM states in mangroves are evolutionarily maintained by natural selection and play a role in expression robustness independent of genetic variation. Transgenerational inheritance of such methylation variation might promote evolvability and facilitate long-term adaptation of extremophiles that have limited genetic diversity.

## Data Availability Statement

The datasets presented in this study can be found in GenBank repository, BioProject PRJNA706926.

## Author Contributions

TT and YW planned and designed the research. YW and YC conducted the experiments. YW, AD, and TT analyzed the data. YW and TT wrote the manuscript. All authors read and approved the submission of the final manuscript.

## Conflict of Interest

The authors declare that the research was conducted in the absence of any commercial or financial relationships that could be construed as a potential conflict of interest.

## Publisher’s Note

All claims expressed in this article are solely those of the authors and do not necessarily represent those of their affiliated organizations, or those of the publisher, the editors and the reviewers. Any product that may be evaluated in this article, or claim that may be made by its manufacturer, is not guaranteed or endorsed by the publisher.

## Footnotes

1
https://phytozome.jgi.doe.gov

2
https://www.R-project.org
