# Supplementary material for: Gene Body Methylation Confers Transcription Robustness in Mangroves During Long-Term Stress Adaptation
Source: Front Plant Sci. 2021 Sep 22;12:733846. doi: 10.3389/fpls.2021.733846 (PMC8493031; doi:10.3389/fpls.2021.733846)
Supplement: Supplementary file 1 [file Image_1.PDF]

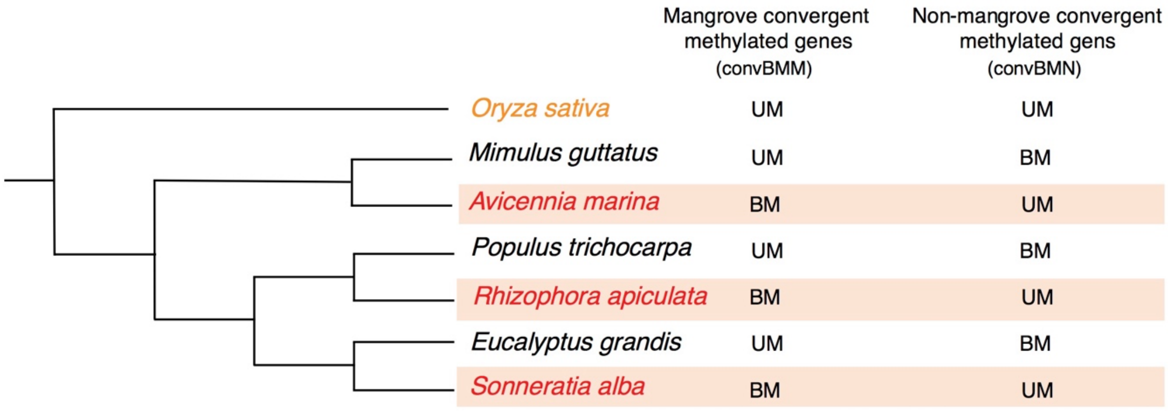

Supplementary Figure 1. Inference of convergent gain of gbM using the convergence at conservative sites method. Convergent evolution is inferred only when all three mangrove or non-mangrove species share the UM status with the outgroup and at least two out of the three species of the other group share the same derived character (methylated state) (based on Xu et al. (2017)). UM, unmethylated genes; BM, body-methylated genes.
