# Supplementary material for: Gene Body Methylation Confers Transcription Robustness in Mangroves During Long-Term Stress Adaptation
Source: Front Plant Sci. 2021 Sep 22;12:733846. doi: 10.3389/fpls.2021.733846 (PMC8493031; doi:10.3389/fpls.2021.733846)
Supplement: Supplementary file 2 [file Image_2.PDF]

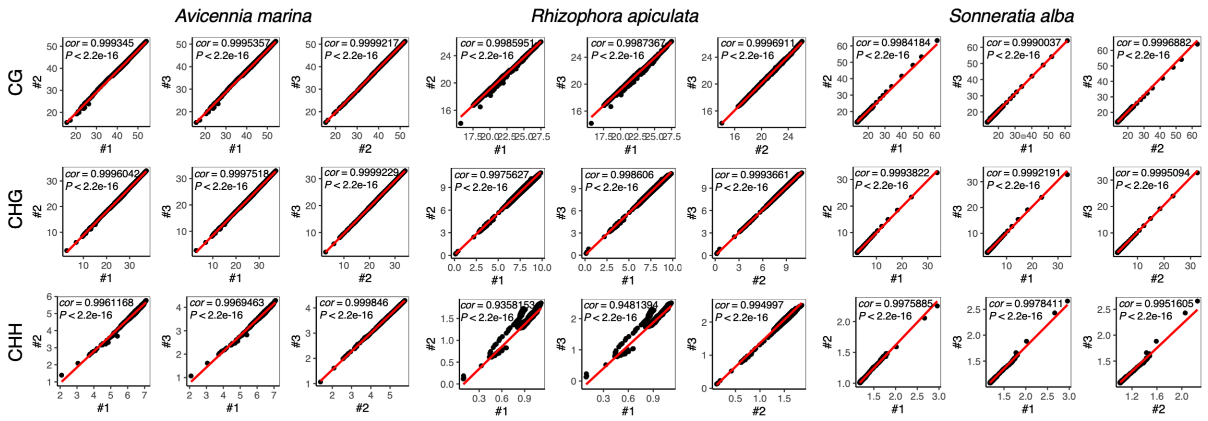

Supplementary Figure 2. Pairwise comparison of DNA methylation levels between biological replicates of *A. marina*, *R. apiculata* and *S. alba*. Sliding window analysis of the methylation levels in all sequence contexts (CG, CHG and CHH) was conducted with window size 100 kb and step size 50 kb. The methylation levels of the 100 kb windows were used to calculate Pearson's correlation coefficient between replicates within speices.
