# Supplementary material for: Gene Body Methylation Confers Transcription Robustness in Mangroves During Long-Term Stress Adaptation
Source: Front Plant Sci. 2021 Sep 22;12:733846. doi: 10.3389/fpls.2021.733846 (PMC8493031; doi:10.3389/fpls.2021.733846)
Supplement: Supplementary file 3 [file Image_3.PDF]

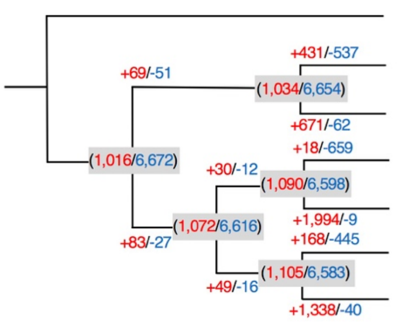

	# of BM	# of UM	rate of gain ($\times 10^{-1}$)	rate of loss ($\times 10^{-1}$)	gain to loss ratio
Setaria viridis	1,293	6,395	-	-	-
Mimulus guttatus	928	6,760	0.065	0.519	0.125
Avicennia marina	1,643	6,045	0.101	0.060	1.682
Populus trichocarpa	449	7,239	0.003	0.605	0.005
Rhizophora apiculata	3,075	4,613	0.302	0.008	36.601
Eucalyptus grandis	828	6,860	0.026	0.403	0.063
Sonneratia alba	2,403	5,285	0.203	0.036	5.615

Supplementary Figure 3. GbM gain and loss across orthologs of mangroves and non-mangrove species using *Setaria viridis* as outgroup. The estimated numbers of gain (red) and loss (blue) of gbM at each internal node are given in parentheses. The estimated numbers of gbM gain and loss in each branch are indicated with plus in red or with minus in blue, respectively. BM, CG body-methylated genes, UM, unmethylated genes.
