# Supplementary material for: Gene Body Methylation Confers Transcription Robustness in Mangroves During Long-Term Stress Adaptation
Source: Front Plant Sci. 2021 Sep 22;12:733846. doi: 10.3389/fpls.2021.733846 (PMC8493031; doi:10.3389/fpls.2021.733846)
Supplement: Supplementary file 4 [file Image_4.PDF]

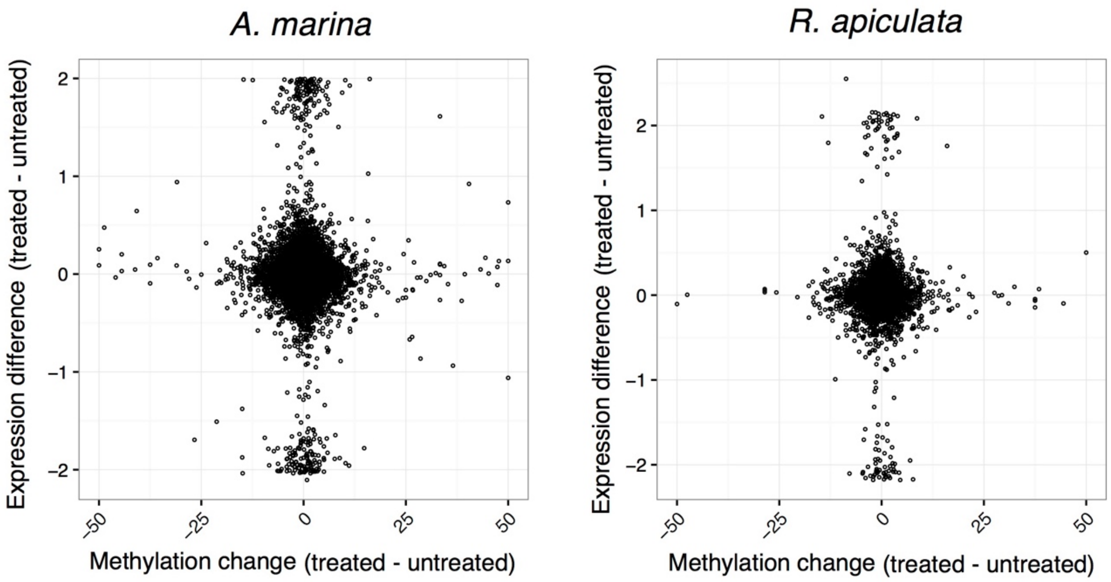

Supplementary Figure 4. Correlation between changes in DNA methylation and changes in gene expression in *A. marina* and *R. apiculata* after salt treatment.
