# Supplementary material for: Gene Body Methylation Confers Transcription Robustness in Mangroves During Long-Term Stress Adaptation
Source: Front Plant Sci. 2021 Sep 22;12:733846. doi: 10.3389/fpls.2021.733846 (PMC8493031; doi:10.3389/fpls.2021.733846)
Supplement: Supplementary file 5 [file Image_5.PDF]

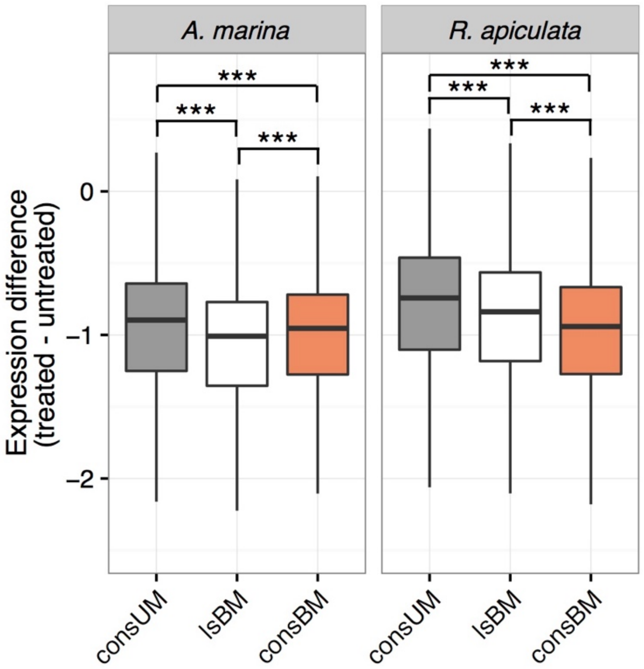

Supplementary Figure 5. Salt-responsive expression differences in relation to gbM conservation. consUM, genes with conserved UM status in all the seven species; lsBM, BM genes with lineage-specific gbM; consBM, BM genes with gbM conserved in at least two species
