# Supplementary material for: Gene Body Methylation Confers Transcription Robustness in Mangroves During Long-Term Stress Adaptation
Source: Front Plant Sci. 2021 Sep 22;12:733846. doi: 10.3389/fpls.2021.733846 (PMC8493031; doi:10.3389/fpls.2021.733846)
Supplement: Supplementary file 6 [file Image_6.PDF]

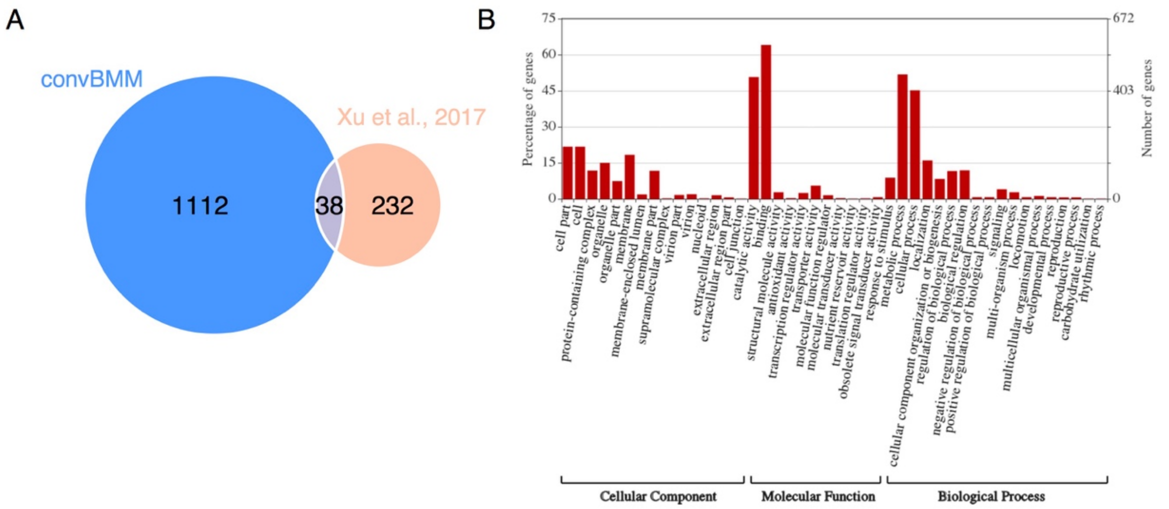

Supplementary Figure 6. Genetic basis and functional annotation of the mangrove convergent methylated genes. (A) Venn diagram shows the overlap between the mangrove convergent methylated genes (convBMM) identified in this paper and genes that underwent convergent sequence evolution identified by Xu et al. (2017). (B) Gene Ontology (GO) analysis of convBMM genes.
