# Supplementary material for: Gene Body Methylation Confers Transcription Robustness in Mangroves During Long-Term Stress Adaptation
Source: Front Plant Sci. 2021 Sep 22;12:733846. doi: 10.3389/fpls.2021.733846 (PMC8493031; doi:10.3389/fpls.2021.733846)
Supplement: Supplementary file 7 [file Table_1.DOCX]

**Supplementary Table 1.** Sample descriptions and alignment statistics.

| Species | Accession | Reference DNA | Uniqe mapped reads | Mapping efficiency (%) | Conversion rate (%) | Coverage |
| --- | --- | --- | --- | --- | --- | --- |
| *A. marina #1*  (100 bp pair-end) | This study | Lambda DNA | 45,223,746 | 84.1 | 99.62 | 10 |
| *A. marina #2*  (150 bp pair-end) |  |  | 64,948,053 | 84.1 | 99.41 | 21 |
| *A. marina #3*  (150 bp pair-end) |  |  | 64,534,876 | 85.6 | 99.39 | 21 |
| *R. apiculata #1*  (100 bp pair-end) | This study | Lambda DNA | 37,409,282 | 69.7 | 99.60 | 15 |
| *R. apiculata #2*  (150 bp pair-end) |  |  | 57,416,573 | 74.1 | 99.52 | 36 |
| *R. apiculata #3*  (150 bp pair-end) |  |  | 52,275,733 | 73.3 | 99.43 | 33 |
| *S. alba #1*  (100 bp pair-end) | This study | Lambda DNA | 48,930,687 | 66.5 | 99.65 | 23 |
| *S. alba #2*  (150 bp pair-end) |  |  | 50,222,153 | 71.0 | 99.46 | 36 |
| *S. alba #3*  (150 bp pair-end) |  |  | 45,395,364 | 72.2 | 99.51 | 33 |
| *O. sativa* | SRR618545 | Chloroplast | 109,918,755 | 50.6 | 97.63 | 6 |
| *M. guttatus* | SRR3286289, SRR3286290, SRR3286291, SRR3286292, SRR3286293, SRR3286294 | Lambda DNA | 38,367,456 | 54.2 | 99.66 | 6 |
| *P. trichocarpa* | SRR3286305 | Lambda DNA | 98,880,728 | 76.1 | 99.70 | 11 |
| *E. grandis* | SRR3286266 | Lambda DNA | 125,835,878 | 61.8 | 99.50 | 9 |
