# Supplementary material for: Gene Body Methylation Confers Transcription Robustness in Mangroves During Long-Term Stress Adaptation
Source: Front Plant Sci. 2021 Sep 22;12:733846. doi: 10.3389/fpls.2021.733846 (PMC8493031; doi:10.3389/fpls.2021.733846)
Supplement: Supplementary file 9 [file Table_3.DOCX]

**Supplementary Table 3.** The comparison of gbM status among paralogs for multi-copy ortholog gene clusters. Gene clusters with only one copy in a species were excluded in that species for this analysis. The proportion of gbM for each gene cluster was calculated as the number of paralogs identified as gbM divided by the total number of genes within that cluster. According to the proportion of gbM, each gene cluster was identified as gbM (=100%), UM (=0% of the paralogs were UM), gbM dominant (>50%, ≠100%), UM dominant (<50%,≠0%), or Balanced (= 50%). Then, the gbM status of each gene cluster was compared with that of the 1:1 ortholog in the same cluster (as shown in the first column).

| Species  (# of multi-copy gene clusters) | Methylation status in ortholog gene cluster | | | | | | |
| --- | --- | --- | --- | --- | --- | --- | --- |
|  | Methylation status in 1:1 ortholog | GbM  (= 100%) | GbM dominant  (> 50%, ≠ 100%) | UM  (= 0%) | UM dominant  (< 50%, ≠ 0%) | Balanced (= 50%) | Differing from the 1:1 ortholog |
| *O. sativa*  (2134) | gbM | 22 | 6 | 0 | 37 | 56 | 37 |
|  | UM | 0 | 4 | 1883 | 78 | 48 | 4 |
| *M. guttatus*  (2021) | gbM | 105 | 46 | 0 | 60 | 131 | 60 |
|  | UM | 0 | 39 | 1347 | 146 | 147 | 39 |
| *A. marina*  (2916) | gbM | 319 | 125 | 0 | 87 | 166 | 87 |
|  | UM | 0 | 27 | 1932 | 147 | 113 | 27 |
| *P. trichocarpa*  (4200) | gbM | 104 | 24 | 0 | 48 | 105 | 48 |
|  | UM | 0 | 7 | 3743 | 99 | 70 | 7 |
| *R. apiculate*  (2228) | gbM | 596 | 82 | 0 | 51 | 208 | 51 |
|  | UM | 0 | 27 | 1075 | 58 | 131 | 27 |
| *E. grandis*  (1940) | gbM | 46 | 22 | 0 | 46 | 101 | 46 |
|  | UM | 0 | 6 | 1483 | 136 | 100 | 6 |
| *S. alba*  (2893) | gbM | 424 | 116 | 0 | 82 | 252 | 82 |
|  | UM | 0 | 47 | 1649 | 146 | 177 | 47 |
