# Supplementary material for: Gene Body Methylation Confers Transcription Robustness in Mangroves During Long-Term Stress Adaptation
Source: Front Plant Sci. 2021 Sep 22;12:733846. doi: 10.3389/fpls.2021.733846 (PMC8493031; doi:10.3389/fpls.2021.733846)
Supplement: Supplementary file 10 [file Table_4.DOCX]

**Supplementary Table 4.** Number of differentially methylated positions (DMPs) induced by salt treatment.

| Species | Sequence context | DMPs | Total sites |
| --- | --- | --- | --- |
| *A. marina* | CG | 84,920 | 13,777,621 |
|  | CHG | 6,226 | 17,826,094 |
|  | CHH | 2,132 | 121,115,317 |
| *R. apiculata* | CG | 30,423 | 7,147,951 |
|  | CHG | 8,792 | 11,012,650 |
|  | CHH | 1,014 | 62,935,185 |
