# Supplementary material for: Gene Body Methylation Confers Transcription Robustness in Mangroves During Long-Term Stress Adaptation
Source: Front Plant Sci. 2021 Sep 22;12:733846. doi: 10.3389/fpls.2021.733846 (PMC8493031; doi:10.3389/fpls.2021.733846)
Supplement: Supplementary file 11 [file Table_5.DOCX]

**Supplementary Table 5.** Salt-responsive methylation changes in genes convergently gaining methylation in mangroves. In each species, we classified all genes into two groups of “Consistent” and “Inconsistent” according to whether their gbM states are consistent between the freshwater-irrigated plants and natural plants or not. BM, body-methylated; UM, unmethylated; convBMM, convergently body-methylated in mangroves; others, other BM or UM genes in separate species.

|  | Species | Methylation type | Gene clusters | gbM status | | % of DMGs | χ^2^ test |
| --- | --- | --- | --- | --- | --- | --- | --- |
|  |  |  |  | Unchanged | Changed |  | (P value) |
| Consistent | *A. marina* | BM | convBMM | 529 | 17 | 3.11 | > 0.05 |
|  |  |  | others | 3144 | 77 | 2.39 |  |
|  |  | UM | convBMM | 468 | 17 | 3.51 | < 0.001 |
|  |  |  | others | 28788 | 149 | 0.51 |  |
|  | *R. apiculata* | BM | convBMM | 982 | 13 | 1.31 | > 0.05 |
|  |  |  | others | 4654 | 87 | 1.84 |  |
|  |  | UM | convBMM | 58 | 2 | 3.33 | < 0.05 |
|  |  |  | others | 19919 | 77 | 0.39 |  |
| Inconsistant | *A. marina* | BM | convBMM | 12 | 19 | 61.29 | > 0.05 |
|  |  |  | others | 191 | 192 | 50.13 |  |
|  |  | UM | convBMM | 11 | 39 | 78.00 | > 0.05 |
|  |  |  | others | 66 | 160 | 70.80 |  |
|  | *R. apiculata* | BM | convBMM | 2 | 0 | 0.00 | > 0.05 |
|  |  |  | others | 158 | 66 | 29.46 |  |
|  |  | UM | convBMM | 38 | 17 | 30.91 | > 0.05 |
|  |  |  | others | 282 | 103 | 26.75 |  |
