# Supplementary material for: Gene Body Methylation Confers Transcription Robustness in Mangroves During Long-Term Stress Adaptation
Source: Front Plant Sci. 2021 Sep 22;12:733846. doi: 10.3389/fpls.2021.733846 (PMC8493031; doi:10.3389/fpls.2021.733846)
Supplement: Supplementary file 13 [file Table_7.DOCX]

**Supplementary Table 7.** Salt-responsive methylation changes for genes undergo sequence convergence (convSeq) in comparison with the others. BM, body-methylated, UM, unmethylated.

| Species | Methylation type | Gene clusters | gbM status | | % of DMGs | χ^2^ test  (P value) |
| --- | --- | --- | --- | --- | --- | --- |
|  |  |  | Unchanged | Changed |  |  |
| *A. marina* | BM | convSeq | 190 | 1 | 0.5 | < 0.001 |
|  |  | others | 3,688 | 304 | 8.2 |  |
|  | UM | convSeq | 188 | 3 | 1.6 | > 0.05 |
|  |  | others | 29,166 | 364 | 1.2 |  |
| *R. apiculata* | BM | convSeq | 187 | 4 | 2.1 | > 0.05 |
|  |  | others | 5,614 | 164 | 2.9 |  |
|  | UM | convSeq | 188 | 3 | 1.6 | > 0.05 |
|  |  | others | 20,151 | 196 | 1.0 |  |
